# Buddhism and Depressive Symptoms among Married Women in Urban Thailand

**DOI:** 10.3390/ijerph17030761

**Published:** 2020-01-25

**Authors:** Ting Xu, Xiaohe Xu, Thankam Sunil, Bangon Sirisunyaluck

**Affiliations:** 1 School of Public Administration, Sichuan University, Chengdu 610065, China; xuting@scu.edu.cn; 2 Department of Sociology, University of Texas at San Antonio, San Antonio, TX 78249, USA; thankam.sunil@utsa.edu; 3 West China School of Public Health, Sichuan University, Chengdu 610065, China; 4 Faculty of Liberal Arts, Maejo University, Chiang Mai 50290, Thailand; poonisa@hotmail.com

**Keywords:** Buddhism, depressive symptoms, mental health, religious involvement, Thailand

## Abstract

A growing body of research has documented salutary associations between religious involvement and poor mental health outcomes, such as depressive symptoms and psychological distress. However, little scholarly attention has been given to the association between Buddhism, a non-Western religious faith, and depressive symptomatology in Thailand. Using random survey data collected from urban Thailand, this study examines the association between religious involvement and depressive symptoms among married women in Bangkok. Findings from multiple linear regression models reveal that (1) Buddhist respondents report significantly lower levels of depressive symptoms than their non-Buddhist counterparts, (2) the frequency of participation in religious activities is significantly and inversely associated with the level of depressive symptoms, and (3) the inverse association between religious participation and depressive symptoms is more salient for Buddhists who frequently practice their faith (i.e., significant interaction effect). Research limitations and directions for future research are discussed.

## 1. Introduction

A growing body of research has documented multifaceted and salutary associations between religious involvement and poor mental health outcomes, such as depressive symptoms and psychological distress in the context of Western societies [1,2,3,4,5,6,7,8]. In spite of inconsistent findings and controversies (e.g., the dark side of religion or the detrimental effects of religious involvement on mental health), scholars of religion and mental health have generally agreed that religion in general and religious involvement in particular, however defined and measured, are protective factors for mental disorders and psychological distress [1,2,3,4,5,6,7,8]. To put it simply, religion benefits mental health, which is defined as “a state of well-being in which the individual realizes his or her own abilities, can cope with the normal stresses of life, can work productively and fruitfully, and is able to make a contribution to his or her community” [9]. However, over the past several decades, limited scholarly attention has been given to the linkage between Buddhism, a non-Western religious faith, and negative mental health outcomes, such as depressive symptomatology in Asian societies (e.g., Thailand, a predominantly Buddhist society). The present study aims to fill this gap in research on religion and mental health.

By analyzing survey data collected from married women in Bangkok, Thailand, this study can make significant contributions to the fields of religion and mental health studies in a number of important ways. First, this study explores the meaning of being self-identified Buddhist and examines the Buddhist doctrines (e.g., the Pāli Canon) for mental health implications. Second, this study investigates the effect of religious practices or participation in religious activities on depressive symptoms among married women in urban Thailand. Third, this study examines the effect of the intersection of Buddhist identity and frequency of participation in religious activities on mental health given that only half (49.4%) of those who are self-identified as Buddhist practice the basic Buddhist doctrines in contemporary Thailand [10]. To the best of our knowledge, these research topics have yet to be systematically investigated.

We begin this study with a brief documentation of the socioreligious context and the landscape of mental health in Thailand. We then review theoretical perspectives and mechanisms that help explain the linkage between Buddhism and mental health outcomes. Next, we quantitatively test whether (1) respondents who are self-identified as Buddhist will report lower levels of depressive symptoms than their non-Buddhist counterparts, (2) the frequency of participation in religious activities will be inversely associated with the level of depressive symptoms, and (3) respondents who are self-identified as Buddhist and actively participate in religious activities (e.g., active Buddhists) will exhibit lower levels of depressive symptoms. We conclude this study by discussing research limitations and promising directions for future research. 

### 1.1. Socioreligious Context and Depression in Thailand

According to the most recent census, Thailand’s population reached 65.98 million in 2010 [11]. While about 40 percent of the Thai population reside in urban (municipal) areas, the majority (60%) reside in rural areas. Bangkok, the capital and most populous city of Thailand, had a population size of 8.3 million, representing 12.6% of the total Thai population. The average educational attainment for the population aged 15 and above was 8.1 years of schooling [11]. 

Theravada Buddhism, constitutionally stipulated by the king, is Thailand’s state religion [12]. The vast majority of the Thai population in the “the Land of Yellow Robes” identified themselves as Buddhist (93.6%), followed by Muslim (4.9%), Christian (1.2%), Hindu (0.06%), Confucian (0.03%), Sikh (0.02%), and other faith tradition or atheist (0.07%) [10]. The 2011 Survey on Status of Society and Culture revealed that although Thailand is a predominantly Buddhist country, only half (49.4%) of those who identified as Buddhist practiced the basic Buddhist doctrines and less than half (40.5%) meditated [10]. With reference to the two fundamental Thai Buddhist practices, 19.1% of those surveyed reportedly offered alms (food) to the Buddhist monks on the daily or weekly basis and 37.1% did so on Buddhist holy/religious days. As for praying, among those who were surveyed, 23.4% prayed on the daily or weekly basis, whereas 22.9% prayed only on Buddhist holy day or during Buddhist lent/religious days. Taken together, these survey results suggest that despite the fact that Thailand is overwhelmingly Buddhist, there are varying degrees of religious involvement among self-identified Buddhists, with only a diminutive percentage adhering to frequent participation in religious activities.

Turning to mental health, about 1.5 million people in Thailand were estimated to live with depression in 2008. Nearly two in three were women [13]. Among major Thai cities, the prevalence of major depression was highest in Bangkok (4.1%) [13]. Depressive symptoms were even more prevalent among women who experienced intimate partner violence in Bangkok [14,15]. However, as of today, poor mental health outcomes such as depression are still significantly under-recognized. Even though mental health has been recently promoted and mental health policies addressing quality and access of care have been improving [16,17], people with mental illness or depression continue to confront stigma [18,19]. Along with healthcare institutions and professionals, Buddhism continues to play an important role in mental health care and treatment, which includes but is not limited to, late pregnancy and postpartum depressive moods, depression due to chronic and acute diseases, and substance abuse or alcoholism [18,19,20,21,22]. Bearing this unique socioreligious context in mind, we investigate the relationship between Buddhism and mental health/depression in urban Thailand.

### 1.2. Theoretical Perspectives

Scholars of religion and mental health have offered numerous theoretical perspectives to explain the salutary effects of various facets or dimensions of religious involvement on mental health. The most noteworthy and relevant to this study are the behavioral perspective advanced by Levin and the stress prevention or reduction perspective articulated by Ellison and his colleagues [1,3,7]. Very briefly, these perspectives posit that religion can improve mental health in three distinct ways. First, religion can improve mental health because religious beliefs often provide meaning and purpose of life, which, in turn, promotes hope, optimism, and positive world views. Second, religion can improve mental health because religion promotes kindness and care and advocates healthy behaviors or lifestyle choices, such as abstinence of tobacco, alcohol, and drug use. Third, religion can improve mental health because frequent participation in religious activities can help establish formal or informal socioreligious networks that provide tangible and nontangible (e.g., emotional) support. Such support can act as a crucial resource for coping in times of emotional disorder or psychological distress [3,7]. As such, religious involvement is positively associated with improved mental health by reducing stressors derived from adverse life events, thereby enhancing individuals’ overall mental health and wellbeing. 

Contrary to the theoretical arguments reviewed above, scholars of religion and mental health have also documented detrimental effects of involvement in religious activities on mental health [4,6]. Two theoretical explanations are advanced by Koenig and Larson (2001) to account for these negative effects [6]. First, individuals who are frequently involved in religious activities but do not conform to certain socioreligious norms may develop a sense of guilt or shame, thus reducing a sense of self-worth and hopefulness, and withdrawing themselves from religious support networks. Second, those who are actively involved in religious activities may use religion to cope with adverse life events or trauma. As such, those who are mentally distressed may turn to religion for comfort, which, in turn, increases religious involvement. It is imperative to point out that these theoretical perspectives and arguments are primarily developed in the context of Western societies and Christianity. In the pages that follow, we apply them to Thailand, a Buddhist society, and critically examine if similar associations between Buddhism and mental health can be established. 

### 1.3. Buddhism and Mental Health

Like other faith traditions, Buddhism can serve as an important meaning-making system that provides multifaceted pathways for enhancing individuals’ mental health by behavior modification and reduction of stressors and depressive symptoms. As revealed in the Pāli Canon, Theravada Buddhism recognizes the existence of mental illness or depressive symptoms. In this religious tradition, mental illness is described as the manifestation of mental defilements or unwholesome states. The symptoms of depression are said to be conditioned, thereby can be identified [23]. Once identified, these symptoms should be removed or reduced [23].

Consistent with the theoretical perspectives considered previously, several Buddhist teachings and concepts provide religious and moral compasses that help Buddhists and lay persons cope with and buffer against stressors, which, in turn, reduces depressive symptoms. For example, karma, described as intentional actions, can affect future lives. As such, avoiding “bad” karma, such as unpleasant, disturbing, or dangerous life events, becomes a life time pursuance for Buddhist believers [18,19]. In addition to avoiding “bad” life events, Buddhism also emphasizes avoidance of taking intoxicants (The Fifth Precept) as they can cloud individuals’ minds. Moreover, merit making, another important Buddhist concept, encourages participation in such religious activities as temple visit, alms offering, and/or becoming a Buddhist monk (for boys). Not only is merit making an indication of commitment to the Buddhist way of life but also a means to overcome “bad” karma [18,19,24]. Finally, the Buddhist principles and teachings are known for promoting compassion, kindness, hope, and optimism [25], albeit in such terms as suffering, which can by exemplified in the Four Noble Truths: “life is filled with suffering; the source of suffering is craving; suffering ends when craving ceases; and the way to end suffering and craving is the eight-fold path” [26]. 

In short, the central tenets of Buddhism provide an important framework for behavior modification, stress coping, and reduction of depression. Given that Buddhism offers an important meaning-making system and pathways to happiness, being a Buddhist might help improve an individual’s mental health. However, this could be true only if the faith is actively practiced. 

### 1.4. Involvement in Religious Activities and Mental Health

Since Buddhism is structured differently as compared with Western faith traditions, there are different types of participation or involvement in religious activities. First, Buddhist believers don’t go to the temple every week. Instead, they visit a temple on a holy day or on the occasion of Buddhist festivals. They may, however, frequently attend Buddhist ceremonies to listen to sermons and make merits. In times of difficulty, Buddhist believers or lay persons in general often turn to the Buddhist monks for explanations, consolation, and religious as well as emotional support [18,19]. Second, Buddhist believers often offer alms to the Buddhist monks in the early morning. Such acts are public demonstrations of faith, commitment to the Buddhist doctrines, adherence to a righteous lifestyle, and hope for a better life in the future [19]. Third, Buddhist believers practice morning and evening chanting or prayers, which can be done at home or in a temple. Engaging in Buddhist chanting or meditation cultivates concentration and positive mindfulness that can facilitate a reduction in the sense of suffering and foster a sense of calm and peacefulness. In fact, akin to meditation, chanting can effectively reduce stress [27]. Finally, Buddhist believers often practice a private Pāli prayer before their bed time to worship the Triple Gems (Triratna): the Buddha, the Dharmma (the Buddha’s teaching), and Sangha (the Noble Order of the Enlightened Followers) [18]. These diverse forms of religious involvement can facilitate positive world views and healthy lifestyles, strengthen emotional attachment and support, and reduce life stresses, which, in turn, can enhance mental health. 

### 1.5. Hypotheses

Based on the literature reviewed above, three research hypotheses are developed.

**Hypothesis** **1** **(H1).** *Respondents who are self-identified as Buddhist will report lower levels of depressive symptoms than their non-Buddhist counterparts.*

**Hypothesis** **2** **(H2).** *The frequency of participation in religious activities will be negatively associated with the level of depressive symptoms.*

**Hypothesis** **3** **(H3).** *The negative association between participation in religious activities and depressive symptoms as specified in H2 will be moderated by respondents’ religious identity. That is, respondents who are self-identified as Buddhist and actively participate in religious activities (i.e., active Buddhists) will exhibit lower levels of depressive symptoms.*

## 2. Materials and Methods

### 2.1. Data and Sample

Data for this study came from a cross-sectional survey conducted in Bangkok, Thailand [28,29]. Consistent with a similar survey conducted in urban China for cross-cultural comparisons [30], the target population was defined as married women residing in Bangkok in 2000. Using a multistage sampling method, the research team reached 1340 married women. In the initial stage, the research team randomly selected five districts from each of the five geographic regions in Bangkok (Northern, Southern, Western, Eastern, and Business-core). This initial random selection resulted in 25 urban districts. In the second stage, the research team randomly selected four blocks from each major street within the selected districts, which gave rise to 204 blocks. In the third stage, the research team randomly selected four housing units from each street block based on block maps. Finally, one married woman from each chosen household was contacted. However, only 816 out of the 1340 Thai wives who were initially contacted agreed to be interviewed. The response rate was 61%. This relatively low response rate was due largely to the sensitive nature of the survey questions pertaining to intimate partner violence and depressive symptoms [28,29]. Of the 816 wives interviewed, five were excluded from this study because of short marital duration (<6 months) and incomplete information. As a result, the final analytic sample for the present study was 811.

The survey instrument was developed in English, then translated into Thai by a bilingual researcher. To ensure the quality and accuracy of the translation, a native Thai translator back-translated the Thai questionnaire into English. This back-translation technique is consistent with research convention in cross-cultural studies [31]. As a standard research protocol, the translated survey instrument was pilot-tested with 20 married Bangkok women living outside of the districts selected for the survey project. After necessary revisions, the finalized instrument was employed for face-to-face interviews conducted by 10 well-trained female professional interviewers. All survey interviews took place in the respondent’s home and lasted, on average, about 40 min. Given the delicate nature of the survey as mentioned previously, a 3-day intensive training workshop was held for the interviewers. The workshop included culturally relevant lectures, best interview practices, and evaluations. The interviewers were instructed to interview the selected respondent when the husband was absent. In the event that an interview began and the husband returned home, the interviewer ended the interview (one such incident occurred and it was subsequently excluded from this study). The survey project was approved by a university’s institutional review board, and informed consent was obtained from all study participants.

### 2.2. Measures

The dependent variable in this study is lifetime depressive symptoms operationalized by the 20-item Center for Epidemiologic Studies Depression Scale (CES-D) [32]. This scale has been previously validated and used in studies conducted in Thailand [14]. Respondents were asked, “Now, tell me how often that you feel about yourself and your life regarding the following statements?”: (1) bothered by things; (2) did not feel like eating; (3) could not shake off the blues; (4) felt just as good as other people; (5) experienced concentration problems; (6) felt depressed; (7) felt everything to be an effort; (8) felt hopeful about the future; (9) felt life had been a failure; (10) felt fearful; (11) experienced restless sleep; (12) felt happy; (13) talked less than usual; (14) felt lonely; (15) felt people were unfriendly; (16) enjoyed life; (17) had crying spells; (18) felt sad; (19) felt disliked by others; and (20) could not get going. Response categories were recoded into 1 = never, 2 = seldom, 3 = sometimes, 4 = often, and 5 = very often with four survey items being reverse-coded. These items were then averaged to create an index variable with greater values representing higher levels of depressive symptoms. The reliability coefficient was 0.81, indicating very good internal consistency. 

To link religious involvement with depressive symptoms, two focal independent variables were used. First, respondents were asked to identify their religious preference or faith tradition. Responses were dummy-coded into 1 = Buddhism and 0 = other faith tradition or no religious preference. Second, respondents were asked how often they participated in religious activities. Responses were reverse-coded into a 5-point Likert scale with 1 = never, 2 = seldom, 3 = sometimes, 4 = often, and 5 = very often. This frequency variable was roughly treated as a metric variable that underlies a continuum of participation in religious activities. 

Several sociodemographic characteristics were included in this study as statistical controls. They were: (1) log-transformed household income, (2) respondent’s age, (3) educational attainment ranging from 1 = less than primary to 7 = higher than college, (4) respondent’s employment status that was dummy-coded into 1 = full time, part time, or retired with unemployed or not working as the reference, (5) number of children, (6) frequency of attendance at secular organizational activities or meetings, and (7) self-reported frequency of alcohol use by respondents ranging from 1 = never to 5 = very often.

### 2.3. Analytic Strategies

Three ordinary least squares (OLS) regression models were estimated to test the research hypotheses specified previously. Net of statistical controls, Model 1 explored if Buddhist respondents reported lower levels of depressive symptoms than their non-Buddhist counterparts (H1). Model 2 examined the hypothesized negative association between the frequency of participation in religious activities and the level of depressive symptoms after controlling for religious preference and sociodemographic characteristics (H2). Model 3 tested the moderating or interaction effect involving Buddhist affiliation and religious participation on depressive symptoms; that is, being active Buddhists can further reduce depressive symptoms (H3). Before conducting these regression analyses, the multiple imputation technique was utilized to replace missing values [33]. The variable of household income exhibited the largest number of missing values (*n* = 20). All analyses were conducted in SPSS Version 26 (IBM, Armonk, NY, USA). 

## 3. Results

### 3.1. Sample Characteristics

Table 1 reports descriptive statistics that summarize respondents’ characteristics. As can be observed from the table, the total number of respondents in this study is 811. The average value for the index of depressive symptoms is 2.51 (ranging from 1.2 to 3.95), indicating that about 50% of respondents (not shown in the table) in this sample reported an average level of depressive symptoms between “seldom” and “sometimes”. In terms of religious involvement, the vast majority of the Thai respondents in this study are Buddhist (90.88%), which is somewhat lower than that (94.6%) reported by the Survey on Status of Society and Culture conducted by the National Statistical Office of Thailand in 2011 [10]. Moreover, the average frequency of participation in religious activities is slightly over 3 (3.06), signifying that respondents “sometimes” participated in religious activities. In regard to basic sociodemographic characteristics, on average married Thai women in this study are 39 years of age with an average education less than high school graduation. As to employment status, about 51% and 18% of respondents are employed full-time and part-time, respectively. While 6% are reportedly not working, 25% are retired. The average number of children reported by respondents is about 2 (1.85). As a measure of civic engagement, respondents reported that they “sometimes” attended non-religious meetings or organized activities outside the home (mean = 2.7). Respondents also reportedly drank alcohol “sometimes” (mean = 1.67 ranging from 1 to 5).

### 3.2. Regression Analyses

Table 2, included below, features the results of OLS regression models to link religious involvement with depressive symptoms in Bangkok, Thailand. There are three notable findings. First, as hypothesized (H1), the regression coefficient for Buddhist in Model 1 is negative and statistically significant at the 0.05 level. This regression result suggests that all else being equal, Buddhist respondents reported significantly lower levels of depressive symptoms than non-Buddhist respondents. Thus, H1 is statistically supported. Second, after controlling for sociodemographic characteristics and religious preference, the frequency of respondents’ participation in religious activities is negatively and significantly associated with the level of depressive symptoms, as shown in Model 2 (*p* < 0.001). More specifically, as the frequency of religious participation increases, the level of depressive symptoms decreases regardless of respondents’ religious preference and sociodemographic backgrounds. This finding lends credence to H2. Third, it is further observed that the interaction term included in Model 3 is highly statistically significant (*p* < 0.001), providing solid support for H3 in that respondents who are self-identified as Buddhist and actively participate in religious activities (i.e., active Buddhists) exhibit lower levels of depressive symptoms. Stated differently, as the frequency of religious participation increases the level of depressive symptoms decrease, and this protective or beneficial effect is more salient for Buddhist respondents, holding sociodemographic characteristics constant. 

Though inconsistent across the three regression models, number of children (*p* < 0.05), frequency of attendance at secular meetings (*p* < 0.05), and alcohol use (*p* < 0.05) emerged as significant predictors of depressive symptoms. Because these variables serve as statistical controls in the present study, no substantive interpretations are rendered.

## 4. Discussion

This study examined the association between religious involvement and depressive symptoms among married women in urban Thailand. As hypothesized, Thai women who were self-identified as Buddhist reported lower levels of depressive symptoms than their non-Buddhist counterparts. This finding is in line with previous studies on Buddhism and depression [18,19,20,21,22]. Resembling research findings from the United States [1,2,3,4,5,6,7,8], this study revealed that the frequency of participation in religious activities was significantly and inversely associated with the level of depressive symptoms. Moreover, Buddhist women who actively practiced their faith exhibited lower levels of depressive symptoms than their Buddhist or non-Buddhist counterparts that were not actively involved in religious activities. It is concluded that Buddhism as a religion of practice can enhance the mental health of married women in urban Thailand. More importantly, the theoretical perspectives on religion and mental health developed in the West can be applied and generalized to Buddhist women in Bangkok. 

While these findings demonstrate salutary effects of Buddhism on depressive symptoms among married women in urban Thailand, there are a few research caveats that are worth noting. First, the measurement of religious participation used in this study did not differentiate between private (e.g., meditation and prayer) and public (e.g., temple visits and attendance at chanting or religious activities) religious participation. Future studies should consider a full array of religious involvement measures, such as frequency of private and public prayers, meditation, temple visit, and/or alms offering. Analyses of these specific types of Buddhist practices can shed new light on the effects of religious involvement on depressive symptoms in a Buddhist context. Moreover, religious salience, religious guidance, and perceptions of divine control that can provide additional insights should be considered for future research. 

Second, the findings reported in this study came from a cross-sectional survey that is twenty years old and does not contain measures with an appropriate temporal order. Therefore, no causal relationships between religious involvement and depressive symptoms are implied. In fact, previous studies suggested that there might be reciprocal relationships between religious involvement and mental health [34]. For example, not only can attendance at religious services prevent the subsequent depressive symptoms, but depressive symptoms themselves can also lead to lower levels of the subsequent attendance at religious services [34]. Nevertheless, more updated longitudinal studies can provide greater accuracy in determining changes in both religious involvement and depressive symptoms over time.

Third, this study exclusively focused on married women in Bangkok. Even though women are more religious and disproportionately affected by mental illness [9], this research focus limits its ability to generalize the findings to other subpopulations in urban Thailand such as married men. For comparative purposes, future research should include both men and women.

Fourth, as reported previously, this study controlled for several significant correlates of depressive symptoms, including number of children, civic engagement (i.e., involvement in secular or social activities), and alcohol use. While these results are congruent with prior research findings [35,36], future research should explore how these significant protective or risk factors, especially alcohol consumption that is strongly and positively associated with depressive symptoms, can be buffered, exacerbated, or strengthened by involvement in religious activities.

Finally, future research may benefit from a qualitative exploration of how involvement in Buddhism is expressed in the context of urban Thailand. While the present quantitative analysis elucidates the association between Buddhism and depressive symptoms among married women in urban Thailand, future research examining both the quantitative and qualitative aspects of Thai religious culture may serve to augment a growing body of research on the association between religion and depressive symptoms in an understudied Buddhist society. To put it simply, a mixed methods design is recommended for future research.

## 5. Conclusions

This study augments the growing evidence for the salutary effects of Buddhism on depressive symptoms in a non-Western society. Specifically, this study reveals protective effects of Buddhist affiliation and frequent participation in religious activities on depression. In addition, the intersection of Buddhist affiliation and religious participation is inversely associated with depressive symptomatology. That is, being active Buddhists significantly reduces levels of depressive symptoms. Taken together, these findings demonstrate the continued importance and relevance of Buddhism in mental health prevention and treatment in urban Thailand.

## Figures and Tables

**Table 1:** Sample characteristics (*n* = 811).

Variables	Mean	SD	*n*	Percent
Dependent variable				
Depressive symptoms (index)	2.51	0.45	-	-
Independent variables				
Buddhist	-	-	737	90.88
Other religions/no religious affiliation (reference)	-	-	74	9.12
Religious participation	3.06	1.02	-	-
Control variables				
Household income (logged)	15.19	4.94	-	-
Age	39.02	10.38	-	-
Educational attainment	3.38	1.89	-	-
Employment full-time	-	-	416	51.29
Employment part-time	-	-	144	17.76
Retired	-	-	201	24.78
Not working (reference)	-	-	50	6.17
Number of children	1.85	1.39	-	-
Meeting attendance	2.70	1.04	-	-
Alcohol use	1.67	0.98	-	-

SD: Standard deviation.

**Table 2:** OLS regression models to predict depressive symptoms.

Variables	Model 1		Model 2		Model 3	
	β	95%CI	β	95%CI	β	95%CI
(1) Household income (log)	−0.002	−0.011 ± 0.007	−0.001	−0.011 ± 0.008	−0.002	−0.011 ± 0.007
(2) Age	−0.003	−0.007 ± 0.001	−0.002	−0.006 ± 0.001	−0.003	−0.006 ± 0.001
(3) Educational attainment	−0.005	−0.024 ± 0.013	−0.006	−0.024 ± 0.013	−0.006	−0.025 ± 0.012
(4) Employment full-time	0.044	−0.105 ± 0.193	0.022	−0.127 ± 0.170	0.022	−0.125−0.170
(5) Employment part-time	0.043	−0.119 ± 0.204	0.029	−0.132 ± 0.190	0.035	−0.125 ± 0.194
(6) Retired	−0.054	−0.194 ± 0.085	−0.057	−0.196 ± 0.082	−0.057	−0.195 ± 0.081
(7) Number of children	0.029	0.001 ± 0.056 *	0.028	0.000 ± 0.055 *	0.027	0.000 ± 0.054 *
(8) Meeting attendance	−0.054	−0.085 ± −0.023 ***	−0.037	−0.069 ± −0.004 *	−0.027	−0.059 ± 0.006
(9) Alcohol use	0.032	0.000 ± 0.065 *	0.031	−0.001 ± 0.064	0.030	−0.002 ± 0.062
(10) Buddhist	−0.112	−0.222 ± −0.003 *	−0.149	−0.261 ± −0.038 **	0.119	−0.066 ± 0.305
(11) Religious participation			−0.055	−0.088 ± −0.021 ***	−0.018	−0.057 ± 0.021
(10) × (11)					−0.259	−0.404 ± −0.115 ***
Constant	2.799	2.562 ± 3.035 ***	2.942	2.691 ± 3.192 ***	2.795	2.533 ± 3.057 ***
*F*	2.813	**	3.540	***	4.337	***
*R^2^*	0.034		0.047		0.062	
*n*	811		811		811	

* *p* < 0.05. ** *p* < 0.01. *** *p* < 0.001. CI: confidence interval.
